# Supplementary material for: Adult re-expression of IRSp53 rescues NMDA receptor function and social behavior in IRSp53-mutant mice
Source: Commun Biol. 2022 Aug 18;5:838. doi: 10.1038/s42003-022-03813-y (PMC9388611; doi:10.1038/s42003-022-03813-y)
Supplement: Supplementary file 2 — Supplementary Information [file 42003_2022_3813_MOESM2_ESM.pdf]

Adult re-expression of IRSp53 rescues NMDA receptor function and social behavior in IRSp53-mutant mice

Young Woo Noh,[1,#] Chaehyun Yook,[2,#] Jaeseung Kang,[2,#] Soowon Lee,[3] Yeonghyeon Kim,[1] Esther Yang,[4] Hyun Kim,[4] and Eunjoon Kim,[1,2,*]

[1]Department of Biological Sciences, Korea Advanced Institute of Science and Technology (KAIST), Daejeon 34141, Korea; [2]Center for Synaptic Brain Dysfunctions, Institute for Basic Science (IBS), Daejeon 34141, Korea; [3]Graduate School of Medical Science and Engineering, KAIST, Daejeon 34141, Korea; [4]Department of Anatomy and BK21 Graduate Program, Biomedical Sciences, College of Medicine, Korea University, Seoul 02841, Korea; [#]These authors contributed equally to the work; *Corresponding author.

Supplementary figures and figure legends

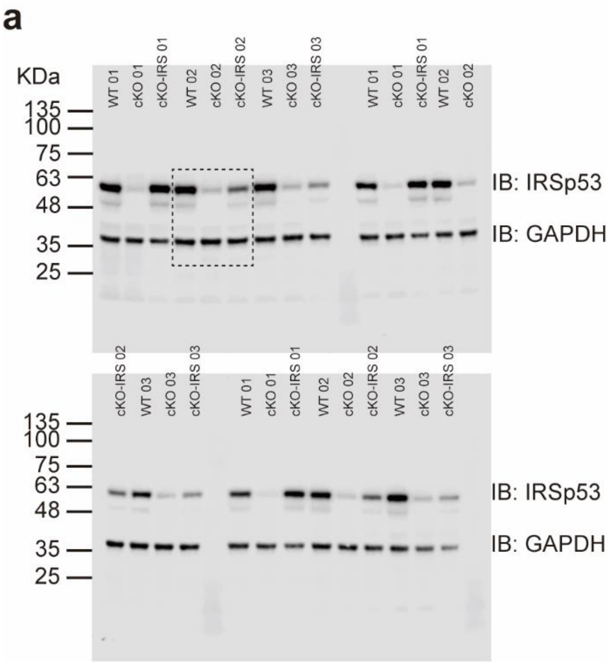

Supplementary Figure 1. Adult re-expression of IRSp53 in WT and IRSp53-cKO mice revealed by immunoblot analysis.

(a) Full-length immunoblot images for IRSp53 proteins expressed in the whole brains of WT, IRSp53-cKO, and PHP.eB-IRSp53-infected IRSp53-cKO mice (WT, cKO, cKO-IRS; 12 weeks), as shown by immunoblotting for IRSp53 and GAPDH (control). The black dashed-line box indicates the portion of the immunoblot used for Figure 1e.

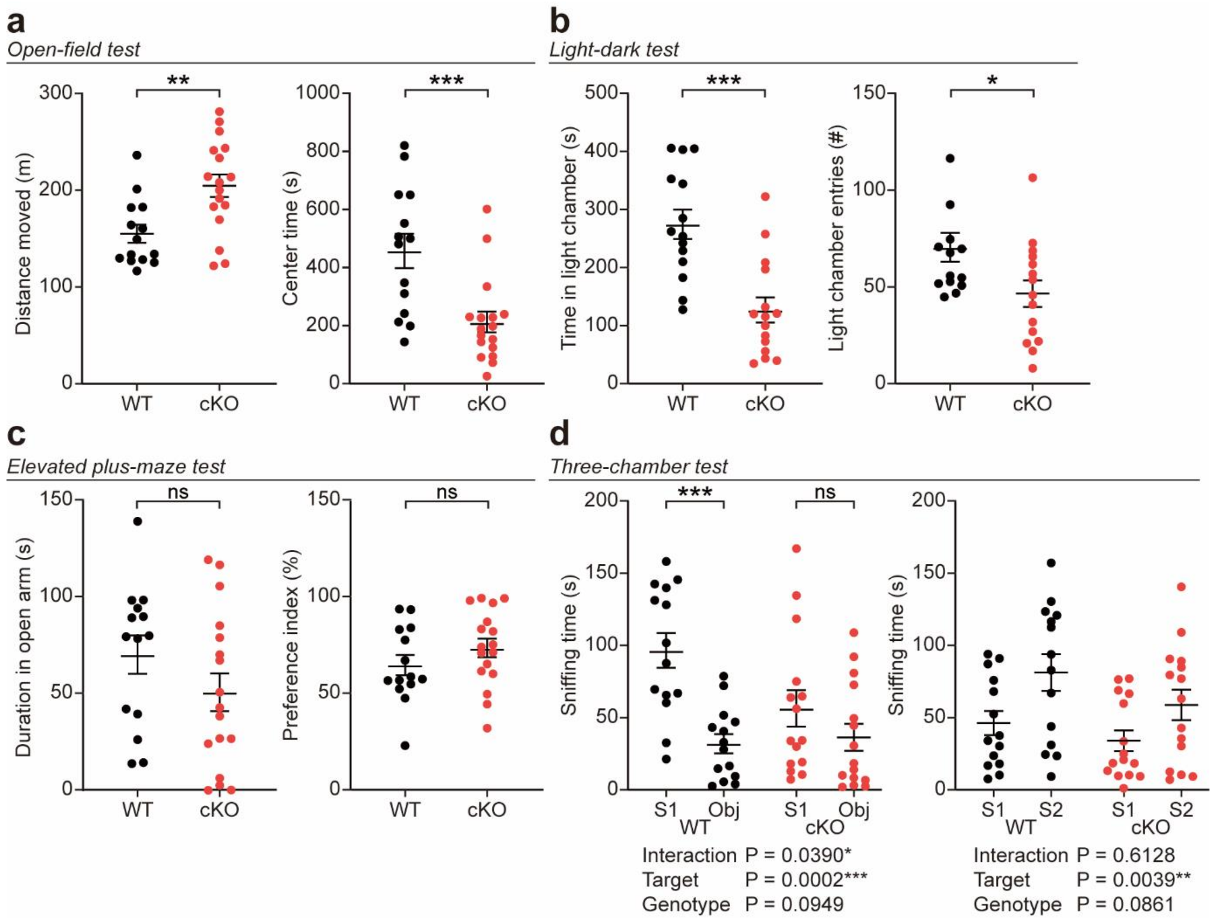

Supplementary Figure 2. Behavioral results from CaMKII-IRSp53-cKO mice.
(a) Hyperactivity and anxiety-like behavior of CaMKII-IRSp53-cKO (*CaMKIIα-Cre;Baiap2*$^{fl/fl}$) mice in the open-field test, as shown by increased locomotor activity and decreased center time. (n = 14 mice [WT], 17 [CaMKIIα-cKO], Student's t-test for distance moved, Mann-Whitney test for center time).
(b) Anxiety-like behavior of CaMKIIα-IRSp53-cKO mice in the light-dark test, as shown by decreased time spent in light chamber and decreased frequency of light-chamber entries. (n = 14 mice [WT], 15 [CaMKIIα -cKO], Student's t-test for time in light chamber, Mann-Whitney test for light chamber entries).
(c) Normal anxiety-like behavior of CaMKIIα-IRSp53-cKO mice in the elevated plus-maze test, as shown by time spent in open arms and preference index (proportion of the difference in time spent in closed and open arms relative to total time). (n = 14 mice [WT], 17 [CaMKIIα -cKO], Student's t-test).
(d) Decreased social interaction but normal social novelty recognition in CaMKIIα-IRSp53-cKO mice in the three-chamber test, as shown by time spent sniffing social and object targets (S1 and O), or novel and familiar social targets (S2 and S1). (n = 14 mice [WT], 15 [CaMKIIα -cKO], two-way ANOVA with Sidak's test).
Significance values are indicated as *p (< 0.05), **p (< 0.01), ***p (< 0.001), or ns (not significant, p > 0.05). Error bars represent the standard errors of means.

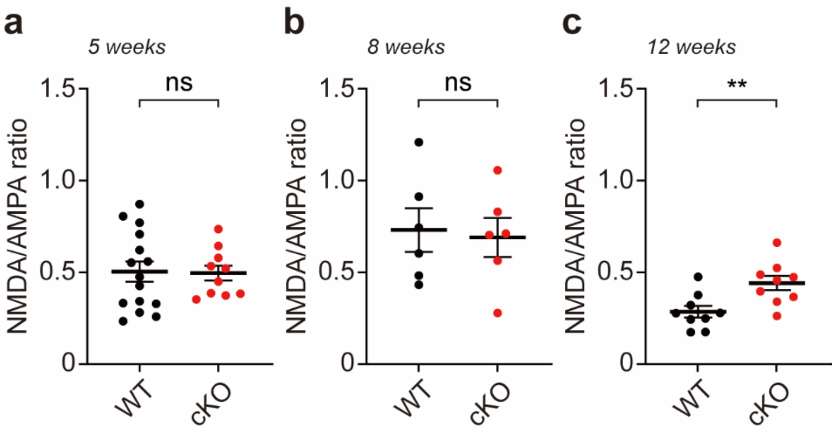

Supplementary Figure 3. Ratios of NMDAR-EPSCs and AMPAR-EPSCs in naïve WT and IRSp53-cKO mice at the age of 5–6, 8, and 12 weeks.

(a) Comparable ratios of NMDAR-EPSCs and AMPAR-EPSCs (NMDA/AMPA ratios) in layer 5 pyramidal neurons from the prelimbic region of the mPFC in naive WT and IRSp53-cKO mice at the age of 5–6 weeks. (n = 15 neurons from 3 mice [WT], 10, 3 [IRSp53-cKO], Student's t-test).

(b) Comparable NMDA/AMPA ratios in layer 5 pyramidal neurons from the prelimbic region of the mPFC in naive WT and IRSp53-cKO mice at the age of 8 weeks. (n = 6, 3 [WT], 6, 3 [IRSp53-cKO], Student's t-test).

(c) Increased NMDA/AMPA ratios in layer 5 pyramidal neurons from the prelimbic region of the mPFC in naive IRSp53-cKO mice, compared with WT mice, at the age of 12 weeks. (n = 9, 3 [WT], 9, 5 [IRSp53-cKO], Student's t-test).

Significance values are indicated as *p (< 0.05), **p (< 0.01), ***p (< 0.001), or ns (not significant, p > 0.05). Error bars represent the standard errors of means.

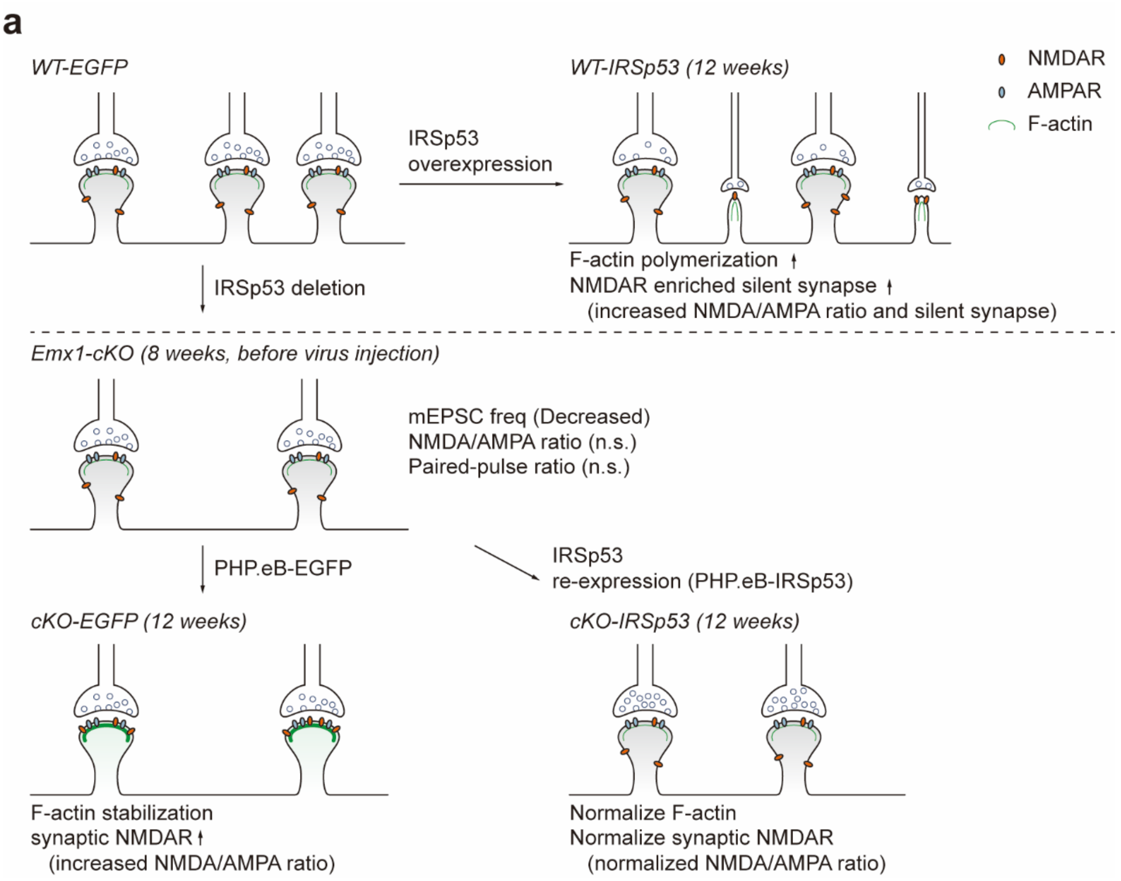

Supplementary Figure 4. A working hypothesis for synaptic changes occurring in IRSp53-cKO mice, IRSp53-overexpressing WT mice, and IRSp53-re-expressing IRSp53-cKO mice.
(a) Deletion of IRSp53 in cortical Emx1-positive layer-5 pyramidal neurons in the mPFC in mice may lead to the initial loss of dendritic spines but no change in the NMDA/AMPA ratio or presynaptic release at around 8 postnatal weeks, based on the normal paired-pulse ratio at ~8 weeks and the decreased mEPSC frequency at ~12 weeks. These changes seem to be followed up, over 8–12 weeks, by secondary changes, including abnormal increases in spine F-actin contents but an increase in the NMDA/AMPA ratio without a change in presynaptic release. Re-expression of IRSp53 in IRSp53-cKO mice starting at 8 weeks may prevent these secondary changes from occurring, although presynaptic release is increased, likely to compensate for the decreased excitatory synapses number. Overexpression of IRSp53 in WT mice starting at 8 weeks may increase F-actin-rich and NMDAR-only silent synapses and redistribute presynaptic proteins from existing synapses to new silent synapses, as supported by increased silent synapses and decreased presynaptic release in existing synapses.
