# Supplementary material for: Adult re-expression of IRSp53 rescues NMDA receptor function and social behavior in IRSp53-mutant mice
Source: Commun Biol. 2022 Aug 18;5:838. doi: 10.1038/s42003-022-03813-y (PMC9388611; doi:10.1038/s42003-022-03813-y)
Supplement: Supplementary file 3 — Description of Additional Supplementary Files [file 42003_2022_3813_MOESM3_ESM.pdf]

Description of Additional Supplementary Files

File name: Supplementary Data 1

Description: The source data behind the graphs in the paper, and statistical results.
